# *Yersinia* effector protein (YopO)-mediated phosphorylation of host gelsolin causes calcium-independent activation leading to disruption of actin dynamics

**DOI:** 10.1074/jbc.M116.757971

**Published:** 2017-03-09

**Authors:** Pavithra Singaravelu, Wei Lin Lee, Sheena Wee, Umesh Ghoshdastider, Ke Ding, Jayantha Gunaratne, Jonathan M. Grimes, Kunchithapadam Swaminathan, Robert C. Robinson

**Affiliations:** From the ‡ Institute of Molecular and Cell Biology, A*STAR (Agency for Science, Technology and Research), Singapore 138673,; § Division of Structural Biology, Wellcome Trust Centre for Human Genetics, University of Oxford, Roosevelt Drive, Oxford OX3 7BN, United Kingdom,; ¶ Diamond Light Source Ltd., Diamond House, Harwell Science & Innovation Campus, Didcot, Oxfordshire OX11 0DE, United Kingdom, and; ‖ Department of Biological Sciences, National University of Singapore, Singapore 117543

**Keywords:** actin, infectious disease, mass spectrometry (MS), phagocytosis, phosphorylation, gelsolin, kinase, Yersinia

## Abstract

Pathogenic *Yersinia* bacteria cause a range of human diseases. To modulate and evade host immune systems, these yersiniae inject effector proteins into host macrophages. One such protein, the serine/threonine kinase YopO (YpkA in *Yersinia pestis*), uses monomeric actin as bait to recruit and phosphorylate host actin polymerization-regulating proteins, including the actin-severing protein gelsolin, to disrupt actin filaments and thus impair phagocytosis. However, the YopO phosphorylation sites on gelsolin and the consequences of YopO-mediated phosphorylation on actin remodeling have yet to be established. Here we determined the effects of YopO-mediated phosphorylation on gelsolin and identified its phosphorylation sites by mass spectrometry. YopO phosphorylated gelsolin in the linker region between gelsolin homology domains G3 and G4, which, in the absence of calcium, are compacted but adopt an open conformation in the presence of calcium, enabling actin binding and severing. Using phosphomimetic and phosphodeletion gelsolin mutants, we found that YopO-mediated phosphorylation partially mimics calcium-dependent activation of gelsolin, potentially contributing to a reduction in filamentous actin and altered actin dynamics in phagocytic cells. In summary, this work represents the first report of the functional outcome of serine/threonine phosphorylation in gelsolin regulation and provides critical insight into how YopO disrupts normal gelsolin function to alter host actin dynamics and thus cripple phagocytosis.

## Introduction

Gelsolin remodels actin organization through Ca^2+^-dependent severing and capping of actin filaments (1). Gelsolin also exists in the blood plasma as an 83-kDa protein, forming part of the extracellular actin-scavenging system. Intracellularly, it is present as an 81-kDa protein in a wide range of cell types (1). It is implicated in various cellular processes, including cell motility, phagocytosis, signaling, apoptosis, cancer, and platelet activation, and it has roles in a number of diseases (2). In familial amyloidosis of the Finnish type, point mutations in gelsolin increase its susceptibility to proteolysis, resulting in amyloid deposits in the eyes, skin, and nerves (3). High expression levels of gelsolin correlate with tumor size, poor patient prognosis, and increased invasive capacity in a range of cancers (4).

Gelsolin has well characterized roles in cell motility and the immune system. Gelsolin-null fibroblasts have pronounced actin stress fibers and migrate slower than the wild-type cells due to aberrant severing and remodeling of actin filaments (5), whereas gelsolin-null mice have multiple defects in morphology and motility in various cell types, including neutrophils, platelets, osteoclasts, and dermal fibroblasts (5). Gelsolin is involved in Fc-γ receptor (FcγR)^6^ and integrin-mediated phagocytosis and is enriched around the phagosome, particularly in IgG-mediated phagocytosis (6), and as such gelsolin-null neutrophils are impaired in FcγR-mediated phagocytosis, whereas gelsolin-null fibroblasts exhibit defective binding and phagocytosis (6). Microbicidal activities of phagocytic cells require chemotaxis and phagocytosis, which involve gelsolin-dependent actin remodeling (7).

Gelsolin comprises six gelsolin homology domains (G1–G6), which, in the absence of calcium, exist in a compact globular arrangement (8). In the presence of micromolar concentrations of calcium, gelsolin undergoes large conformational changes to adopt an open conformation in which the 47-residue-long linker between G3 and G4 extends, allowing the two halves (G1-G3 and G4-G6) to bind the barbed ends of actin filaments, exposing the major actin-binding surfaces on G1, G2-G3, and G4 (9, 10). Activated gelsolin binds to the side of an actin filament and severs that filament into two smaller polymers while capping the barbed end of one-half of the severed filament. The binding of polyphosphoinositides (PPIs) to gelsolin (particularly PIP_2_) uncaps gelsolin from the barbed end to re-enable growth of the actin filament (11). Besides regulation by calcium and PIP_2_, gelsolin can also be regulated by pH, proteolysis, and phosphorylation. Gelsolin undergoes calcium-independent activation below pH 6 (12). Caspase-3, a critical mediator of apoptosis, cleaves gelsolin in the linker region between domains 3 and 4, giving rise to an N-terminal gelsolin fragment (residues 1–352) that is pro-apoptotic (13). Gelsolin is phosphorylated by the tyrosine kinase c-Src primarily at Tyr-438 in the domain G4. Artificial phosphorylation of plasma gelsolin at its N terminus removes its dependence on calcium activation (14). In osteoclasts, proline-rich tyrosine kinase 2 (PYK2) phosphorylates gelsolin on tyrosine residues, reducing gelsolin-actin interactions while increasing the association of gelsolin with PIP_2_ (15). Our recent work has shown that YopO kinase from *Yersinia* phosphorylates human gelsolin through a mechanism that involves the formation of a ternary complex with actin (16).

*Yersinia* bacteria cause a range of human diseases, including gastrointestinal syndromes (*Yersinia enterocolitica*), Far East scarlet-like fever (*Yersinia pseudotuberculosis*), and the plague (*Yersinia pestis*). Pathogenic *Yersinia* species use a type III secretion system to inject effector proteins (Yops) into host macrophages. These comprise six proteins, YopO/YpkA, YopH, YopM, YopE, YopT, and YopJ, that collectively inhibit phagocytosis and reorient the host immune responses (17). YopO, also known as YpkA in *Y. enterocolitica,* harbors a serine/threonine kinase domain (18) and a guanidine nucleotide dissociation inhibitor (GDI)-like domain (19). The kinase activity of YopO is activated in the host cytosol upon binding to actin (20). Actin becomes sandwiched between the serine/threonine kinase domain and the GDI-like domain, which stabilizes the kinase active site in an active conformation (16). Actin-binding proteins are recruited to YopO-actin through the bound actin to form ternary complexes, some of which are phosphorylated by YopO, including Ena/VASP family proteins, DIAPH1, WASP, and gelsolin (16). However, the phosphorylation sites on the phosphorylated substrates and their consequences on actin remodeling have yet to be examined.

In this study, we determined the functional implications of gelsolin phosphorylation by YopO. We identified the phosphorylation sites on gelsolin by mass spectrometry and validated these sites by *in vitro* phosphorylation assays. Molecular dynamics simulations on the modeled phosphorylated gelsolin indicate that phosphorylation by YopO contributes to the instability of inactive gelsolin. Furthermore, actin polymerization assays of phosphomimetic mutants of gelsolin confirm that phosphorylation by YopO confers on gelsolin Ca^2+^-independent activation. Thus, these findings suggest how YopO may circumvent the native calcium-signaling pathways for gelsolin activation to manipulate the host actin cytoskeleton and resist phagocytosis.

## Results

### Gelsolin is phosphorylated between G3 and G4

**Figure 1.**
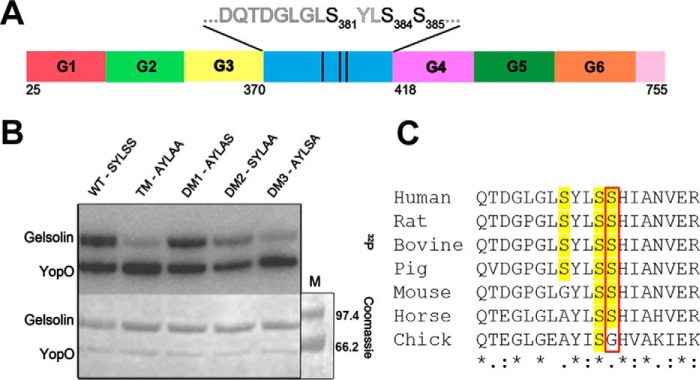
**YopO phosphorylates gelsolin in the linker region between G3 and G4.** *A,* domain architecture of human cytoplasmic gelsolin with identified phosphorylation sites (indicated in *bold*). *B, in vitro* phosphorylation of gelsolin mutants by YopO. YopO WT (2 μm) was incubated with Sf9 actin (2 μm) and gelsolin mutants (12 μm) in the presence of [γ-^32^P]ATP in kinase buffer. Proteins were separated by SDS-PAGE and visualized by Coomassie Blue staining (*bottom panel*). *M* denotes the molecular weight marker. Destained gels were dried and exposed to X-ray film (*top panel*). *C,* sequence alignment of human gelsolin residues 374–392 against gelsolin from different species, highlighting the conservation of Ser-385 (*red box*) in human, mouse, rat, pig, horse and bovine, but not chicken).

To identify the YopO phosphorylation sites on gelsolin, we carried out an *in vitro* phosphorylation assay of gelsolin by YopO in the presence of actin and analyzed the modifications by LC-MS/MS. Phosphorylation sites were identified at Ser-381, Ser-384, and Ser-385 (numbering corresponds to plasma gelsolin). These residues lie in the linker region between the gelsolin domains G3 and G4 (Fig. 1*A* and supplemental Fig. S1). To validate these sites, phospho-deletion mutants of gelsolin, in which the respective serine residues were mutated to alanine, were generated (supplemental Table S1) and tested for radioactive γ-^32^P incorporation in the presence of YopO and Sf9 actin. In comparison with wild-type (WT) gelsolin, the triple mutant TM (^381^AYLAA^385^) displayed the least phosphorylation, and the double mutant DM3 (^381^AYLSA^385^) had a similar level of phosphorylation as TM (Fig. 1*B* and supplemental Fig. S2). Both TM and DM3 possess mutations at positions 381 and 385, indicating that YopO readily phosphorylates these residues in WT gelsolin. The double mutant DM2 (^381^SYLAA^385^) showed a significant reduction in phosphorylation, whereas DM1 (^381^AYLAS^385^) had only a slight attenuation. These data suggest that Ser-385 is the major phosphorylation site, based on the reduction in incorporation of radioactivity observed for the mutants TM and DM3, whereas Ser-381 and Ser-384 are minor phosphorylation sites. Sequence alignments of gelsolin across species revealed the conservation of Ser-385 across human, mouse, rat, pig, horse, and bovine but not in chicken (Fig. 1*C*). Chickens are rarely transmission hosts for *Yersinia* (21).

### Phosphorylation may induce structural instability in inactive gelsolin

**Figure 2.**
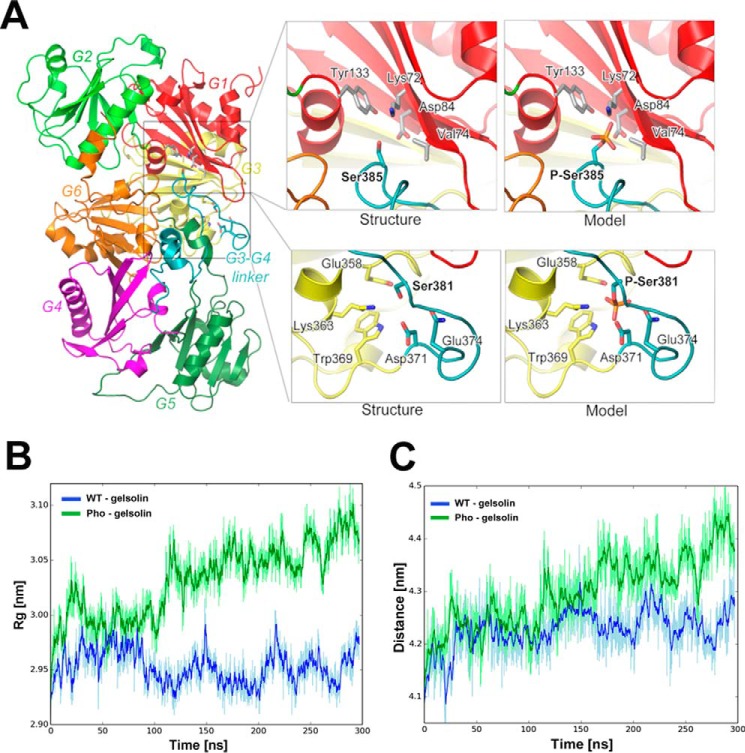
**Molecular dynamics simulations of the effects of phosphorylation by YopO on the gelsolin structure.** *A,* structure of inactive gelsolin showing Ser-381 and Ser-385 and surrounding residues (as *sticks*) alongside the models of the phosphorylated state. *B*, change in the radius of gyration (*R_g_*) of WT and phosphorylated gelsolin during MD simulations. In the case of WT, the simulation stabilizes after 50 ns. In contrast, for phosphorylated gelsolin, the *R_g_* continues to increase throughout the course of simulation, indicating an opening of the gelsolin structure, which is a hallmark of its activation. *C*, evolution of the distance between the center of mass of N- and C-terminal halves of gelsolin during the MD simulation. In the case of WT gelsolin, the distance stabilized after an initial increase of 0.15 nm during the first 50 ns of the simulation. For phosphorylated gelsolin, the distance increased linearly with time, suggesting an opening of its inactive compact arrangement.

To obtain insight into the structural implications that could arise from phosphorylation at Ser-381 and Ser-385, we modeled their positions in the crystal structure of full-length inactive gelsolin (Fig. 2*A*) (PDB code 3FFN (22)). The residues that lie in the vicinity of these serines, as derived from the inactive structure of full-length human cytoplasmic gelsolin (PDB code 3FFN), are tabulated in supplemental Table S2. Ser-381 packs against G3, lying in close proximity to the negatively charged Glu-358 and bulky Trp-369 on G3 at distances of 3.8 and 4.7 Å, respectively. Ser-381 also lies in close proximity to the negatively charged residues Asp-371 and Glu-374 on the G3-G4 linker, at distances of 2.9 and 4.2 Å, respectively. Likewise, Ser-385 packs against G1 and lies in close proximity to the charged residues Lys-72 (4.3 Å) and Asp-84 (6.3 Å) and hydrophobic residues Tyr-133 and Val-74 in G1. We hypothesized that the introduction of negative charges by phosphorylation, coupled with steric hindrance from the much bulkier phosphate groups, may induce repulsion of G1 and G3. To test this hypothesis, we carried out molecular dynamics simulations with calcium-free WT gelsolin and the phosphorylated model (based on PDB code 3FFN) to verify that phosphorylation of these sites results in instability to the compact form of calcium-free inactive gelsolin. Although WT gelsolin remained stable for 250 ns following a 50-ns equilibration, gelsolin that has been phosphorylated at Ser-381, Ser-384, and Ser-385 consistently increased its radius of gyration (*R_g_*) (Fig. 2*B*), and the distance between the centers of the masses of G1–G3 and G4–G6 during this time period (Fig. 2*C*), indicating a destabilizing effect by phosphorylation on the inactive conformation. Size-exclusion chromatography coupled with multiangle light scattering experiments confirmed that the phosphomimetic triple mutant (PM5, S381D/S384D/S385D), where the serines were mutated to aspartic acids to mimic phosphorylation, had higher *R*(avg) (geometric and hydrodynamic radius) than WT gelsolin, supporting the MD simulation data that predicted that the phosphorylated form of gelsolin adopts a more extended conformation than the WT (supplemental Table S3).

### Phosphorylation activates gelsolin's severing activity

**Figure 3.**
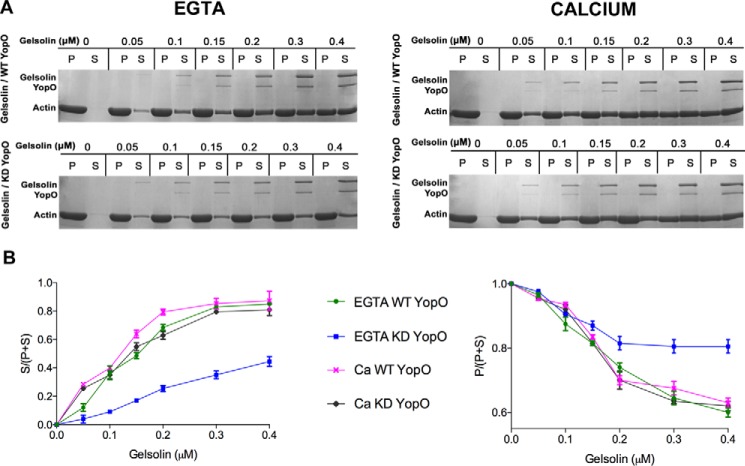
**YopO-phosphorylated gelsolin severs F-actin under EGTA conditions.** *A*, sedimentation assay on *in vitro*-phosphorylated gelsolin under EGTA and calcium conditions. Polymerized F-actin (4 μm) was incubated with various concentrations of phosphorylated gelsolin (*gelsolin/WT YopO*) or non-phosphorylated gelsolin (*gelsolin/KD YopO*) in 1.5 mm EGTA or 1 mm CaCl_2_ for 1 h at room temperature and subjected to ultracentrifugation. Supernatants (*S*) and pellets (*P*) were analyzed by SDS-PAGE. Redistribution of actin from the pellet to the soluble fraction, indicative of severing and/or sequestration, is observed for phosphorylated gelsolin. *B*, percentages of actin in the supernatant fractions (*left*) and pellet fractions (*right*) were plotted as a function of the total concentration of gelsolin in the presence and absence of EGTA conditions. Data represent the average of three experiments.

**Figure 4.**
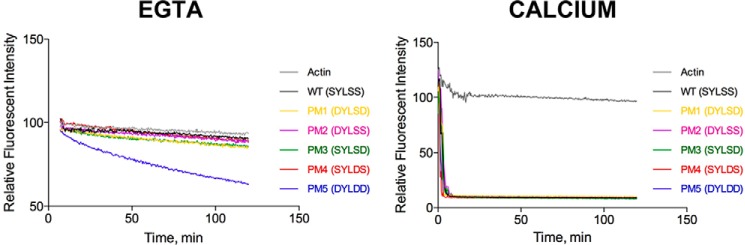
**Pyrene actin depolymerization assay with gelsolin phosphomimetic mutants in EGTA conditions.** Polymerized F-actin (12 μm) was incubated with different gelsolin mutants (6 μm) in 1.5 mm EGTA or 1.0 mm calcium. The loss of pyrenyl fluorescence was measured. In EGTA, the triple mutant PM5 is more active at severing actin filaments, compared with WT and the other mutants. In calcium, phosphomimetic mutants and WT depolymerize actin filaments to similar extents.

To test the effect of phosphorylation on the gelsolin-actin interaction, *in vitro*-phosphorylated gelsolin was tested for its ability to sever actin filaments via a sedimentation assay. Under calcium-free conditions, gelsolin, phosphorylated by YopO WT, was active in severing actin filaments, as judged by the appearance of actin in the soluble fraction, compared with the non-phosphorylated gelsolin, across a range of gelsolin concentrations (Fig. 3*A*). In the presence of calcium (1.0 mm), both phosphorylated and non-phosphorylated gelsolin was observed to sever actin to a similar extent as revealed by comparing the ratios of actin in the soluble *versus* pellet fractions (*Ca WT YopO versus Ca KD YopO*) (Fig. 3*B*). In an attempt to validate these results, a more extensive set of phosphomimetic mutants of gelsolin were created, where the serines were mutated to aspartic acid as follows: PM1 (S381D/S385D); PM2 (S381D); PM3 (S385D); PM4 (S384D); and PM5 (S381D/S384D/S385D). We measured the decrease in pyrenyl fluorescence of F-actin to compare the activities of WT gelsolin and phosphomimetic mutants on the depolymerization of actin. At saturating calcium concentrations (1.0 mm), both WT and the phosphomimetic mutants exhibit similar depolymerizing kinetics (Fig. 4). However, in calcium-free conditions (1.5 mm EGTA), WT gelsolin remained inactive, yet the triple phosphomimetic mutant (PM5, S381D/S384D/S385D) displayed pronounced actin depolymerization activity.

**Figure 5.**
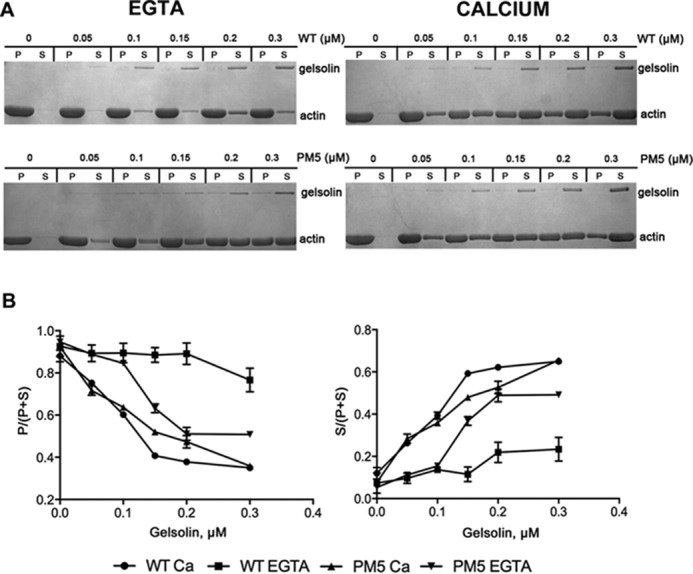
**Sedimentation assay on gelsolin WT and the phosphomimetic mutant PM5.** *A*, 4 μm F-actin was incubated with different concentrations of gelsolin WT or PM5 (0–0.3 μm) for 1 h at room temperature and subjected to ultracentrifugation, and the supernatants (*S*) and the pellets (*P*) were analyzed by SDS-PAGE. *Left,* 1.5 mm EGTA. *Right,* 1 mm calcium chloride. In the EGTA conditions, PM5 severs actin filaments to a greater extent compared with WT. However, at high calcium levels, both WT and PM5 sever filaments to similar extents. *B*, percentages of actin in the pellet fractions (*left*) and supernatant fractions (*right*) were plotted as a function of the total concentration of gelsolin in the presence and absence of EGTA. Data represent the average of three experiments. PM5 severs actin filaments to a greater extent, compared with WT in EGTA conditions. In contrast, at high calcium levels, both WT and PM5 sever filaments to the same extent.

Following this, the severing activities of WT gelsolin and PM5 were compared in an actin sedimentation assay (Fig. 5*A*). At saturating calcium levels (1.0 mm), PM5 severed and distributed actin into a soluble fraction similar to WT gelsolin, across a range of protein concentrations. However, in the absence of calcium, although WT gelsolin did not sever actin significantly, a substantial decrease in the pelletable actin fraction and a corresponding increase of actin in the supernatant were observed for the mutant PM5 (S381D/S384D/S385D) across the tested concentrations of gelsolin. Furthermore, PM5 appears to partition into the pellet fraction and co-sediment with F-actin, which may suggest that PM5 (in EGTA) preferentially severs and caps F-actin over sequestering G-actin. A slight redistribution of actin into the soluble fraction was detected with WT gelsolin in calcium-free conditions, possibly due to the residual activity of gelsolin. The proportion of actin in the pellet or soluble fraction in the presence of different concentrations of gelsolin is plotted in Fig. 5*B*. These data demonstrate that the incorporation of acidic residues into the linker region of gelsolin either reduces or removes its calcium requirement for activity.

**Figure 6.**
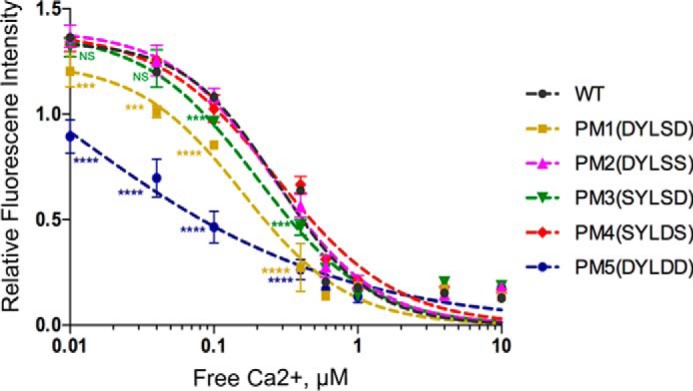
**Pyrene actin depolymerization assay using gelsolin phosphomimetic mutants at different Ca^2+^ concentrations.** Polymerized F-actin (12 μm) was incubated with different gelsolin mutants (6 μm) at a molar ratio of 2:1, and depolymerization was observed after 3 h by titrating against EGTA-buffered calcium levels. PM1 and PM5 require lower calcium levels to sever the filaments, in comparison with the other mutants and WT. Data represent the average of six measurements from two independent experiments in which each sample was set up and measured in triplicate. Data were fitted with Hill equation to obtain the *K_d_* values of WT and different gelsolin mutants. *p* values are expressed relative to WT (***, *p* < 0.001; ****, *p* < 0.0001, *NS,* non-significant) as determined by one-way analysis of variance with Bonferroni post-tests, *n* = 6.

These data prompted us to further investigate the activities of the phosphomimetic mutants at different calcium concentrations (Fig. 6). A pyrene actin depolymerization assay was carried out against different calcium concentrations, in which the pyrene fluorescence was measured after 3 h upon addition of the various gelsolin constructs to F-actin. The value along the *y* axis is the measure of the fraction of F-actin remaining in the sample upon depolymerization by gelsolin. The *y* axis represents the percentage of actin that remains as F-actin following depolymerization by gelsolin. A fit to the data with the Hill equation (Fig. 6) yields *K_d_* values for the calcium requirement for gelsolin activity of 0.37 μm for WT, 0.23 μm for PM1, 0.33 μm for PM2, 0.27 μm for PM3, 0.36 μm for PM4, and 0.11 μm for PM5, suggesting that PM5 (S381D/S384D/S385D) and PM1 (S381D/S385D) were substantially more active at depolymerizing actin than WT or other mutants at lower calcium concentrations. At intermediate concentrations of calcium, there is a small but significant increase in activity for PM3. At calcium concentrations above 0.7 μm, the activities of WT and phosphomimetic mutants were indistinguishable, agreeing with the depolymerization kinetics (Fig. 4). These biochemical results, together with structural modeling, suggest that the introduction of negative charges and the steric hindrance destabilizes the inactive closed conformation of gelsolin, paralleling calcium activation, where the binding of calcium destabilizes the inactive conformation. Because gelsolin is phosphorylated by YopO in its active actin-bound form, we hypothesize that the phosphorylated gelsolin will not be able to revert to the inactive conformation, thus remaining constitutively active.

### Gelsolin phosphomimetic mutants display reduced nucleation activity

**Figure 7.**
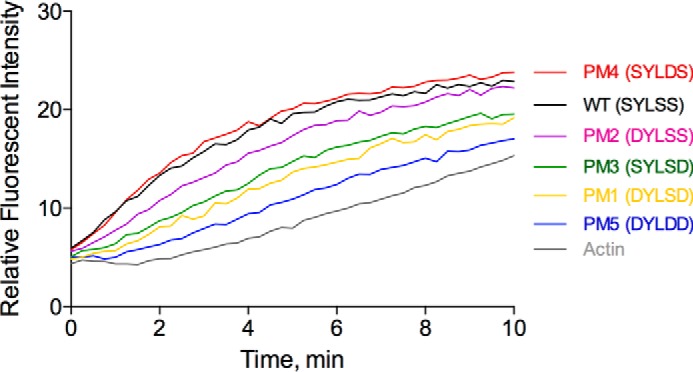
**Actin nucleation assay with gelsolin phosphomimetic mutants.** G-actin was incubated with different gelsolin mutants at a molar ratio of 40:1 for 1 h at 0.1 mm CaCl_2_ followed by the addition of 10× KMEI buffer. PM5 nucleated actin filaments less efficiently in comparison with WT. Data represent the average of three experiments.

Next, we compared the actin nucleation activity of the phosphomimetic mutants in comparison with WT gelsolin (Fig. 7). In the absence of profilin, gelsolin nucleates actin filament formation by binding to two actin monomers to form an actin filament nucleus (23). The actin filament nucleation properties of the WT and mutants were examined via a pyrene-actin polymerization assay at a gelsolin to actin ratio (1:40) in the presence of Ca^2+^ (0.1 mm). Ca^2+^ concentrations above 1 μm are required for gelsolin to bind to two actin monomers via its two halves G1–G3 and G4–G6 to form a stable actin nucleus (8). Although WT gelsolin eliminated the lag phase and increased the initial rate of polymerization, four of the five phosphomimetic mutants exhibited reductions in nucleation activity. Nucleation was most strongly attenuated in PM5 (S381D/S384D/S385D) followed by the PM1 (S381D/S385D), PM3 (S385D), and PM2 (S381D). PM4 (S384D) was indistinguishable from WT. These data suggest that the substitution of the phosphorylation sites by aspartic acid, which partially mimics phosphorylation, lowers the *in vitro* nucleation activity, possibly by inducing a change in the G3–G4 linker to a conformation that is suboptimal for forming a stable actin nucleus with G1–G3 and G4–G6.

## Discussion

Previously, we demonstrated that YopO uses actin as bait to recruit and phosphorylate actin-filament-regulating proteins (16). YopO binds to subdomain 4 of actin, leaving the common binding pocket between subdomains 1 and 3 of actin available, a region of interaction for the majority of actin-binding proteins. A subset of actin filament-regulating proteins, those that are sufficiently elongated to span from the actin-binding site to the kinase catalytic cleft and can present suitable residues, are phosphorylated by YopO, including gelsolin, mDia1, VASP, EVL, and WASP (16, 25). Here, we have mapped the YopO phosphorylation sites on the substrate gelsolin via proteomic analysis and have determined the effects of phosphorylation on the activities of gelsolin in actin filament dynamics.

We identified Ser-385 as the major YopO phosphorylation site on gelsolin. YopO-phosphorylated gelsolin has actin-severing activity in the presence of EGTA. To validate the effect of phosphorylation at these sites, phosphomimetic mutants were created to mimic the phosphorylated state of gelsolin. Although WT gelsolin remains inactive in calcium-free conditions, the phosphomimetic mutants exhibit varying degrees of activity in depolymerization and severing actin filaments in both sedimentation and pyrene-actin assays. Mutants incorporating both S381D and S385D, PM5 (S381D/S384D/S385D), followed by PM1 (S381D/S385D), display the strongest activity in calcium-free conditions. However, single phosphomimetic mutants did not display any activity in EGTA conditions, probably due to the inability of substitution by aspartic acid to completely mimic phosphorylation. Nevertheless, phosphomimetic mutants PM1 (S381D/S385D), PM2 (S381D), PM3 (S385D), and PM5 (S381D/S384D/S385D) exhibit reduction in nucleation activity. Actin nucleation occurs through binding to two actin monomers in an appropriate orientation (26), which in the case of gelsolin involves the linker between G3 and G4 wrapping around the filament (8). The reduction in nucleation by phosphorylated gelsolin may be due to the interference of the phosphorylated sites in adopting a conformation that is optimal for actin nucleation. However, inhibiting nucleation by gelsolin is unlikely to have a physiological role *in vivo*, because pointed-end elongation is not favored in the presence of profilin *in vivo*. The identification of phosphorylation sites within the linker region of gelsolin and its effects on gelsolin activity support a role for the linker in regulating the activity of gelsolin. YopO phosphorylation sites Ser-381, Ser-384, and Ser-385 lie in close proximity to a minor Src phosphorylation site Tyr-382 (Tyr-409 in cytoplasmic gelsolin) (27), caspase-3 cleavage site Asp-376 (Asp-403 in cytoplasmic gelsolin) and granzyme cleavage sites Asp-376 and Leu-383 (Asp-403 and Leu-410, respectively, in cytoplasmic gelsolin) (supplemental Table S4) (28), suggesting that phosphorylation of gelsolin by YopO could interfere with the regulation of gelsolin by these enzymes.

### Functional relevance of YopO phosphorylation sites on gelsolin

To explore the possibility that YopO phosphorylation sites on gelsolin are biologically relevant but have yet to be experimentally reported, we used the phosphorylation site predictor tool in PHOSIDA (29) to predict phosphorylation sites on gelsolin. PHOSIDA uses a support vector machine built on a database of reported phosphorylation sites and encompasses structural information and conservation of phosphorylation sites across species to predict further phosphorylation sites. Biologically important phosphorylation sites are evolutionarily constrained due to their key roles in regulating protein function (30, 31). The prediction results were consistent with the experimental data (supplemental Table S5) with a precision cutoff at 100%, and PHOSIDA predicted two phosphorylation sites on gelsolin, Ser-384 and Ser-385, agreeing with our identification of Ser-385 as the major phosphorylation site of YopO on gelsolin and Ser-384 as the minor site. With the use of phosphomimetic mutants, we have validated that the YopO phosphorylation sites on gelsolin affect actin dynamics. The functional relevance and evolutionary conservation of these sites suggest that phosphorylation at these sites is biologically relevant and is likely involved in native phospho-regulation of gelsolin by a yet to be identified kinase(s). These data support the hypothesis that YopO targets native kinase phosphorylation sites and serves as a useful tool to probe native phospho-regulation.

### Contribution to actin filament disruption

**Figure 8.**
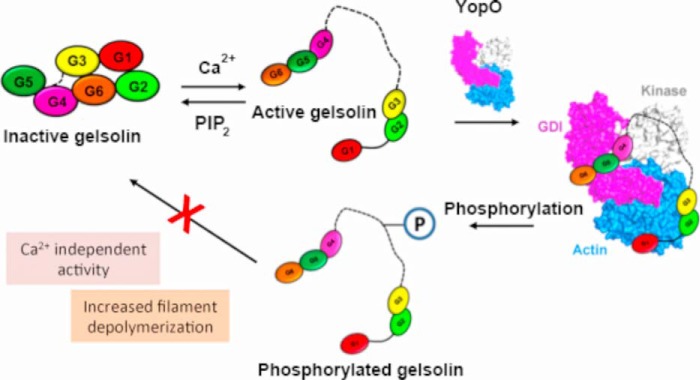
**Model for the implications of phosphorylation by YopO on native gelsolin regulation.**

This work provides insight into how phosphorylation of gelsolin perturbs cytoskeletal assembly and remodeling in phagocytosis. Here, we propose a model by which YopO (Fig. 8), via its kinase activity, manipulates gelsolin leading to misregulated actin remodeling that perturbs phagocytosis. YopO phosphorylation allows gelsolin to acquire severing and depolymerization activity in the absence of calcium. Furthermore, phosphorylated gelsolin may be incapable of resuming its inactive compact arrangement, interfering with native cellular regulation. As such, YopO may reduce cellular F-actin through misregulated activation of gelsolin by increasing gelsolin-induced actin depolymerization and severing. As gelsolin is involved in FcγR and integrin-mediated phagocytosis (6), this work demonstrates how YopO, through its effects on gelsolin, may attenuate macrophage phagocytosis.

## Materials and methods

### Expression and purification of proteins

*Y. enterocolitica* YopO WT and mutants and human gelsolin were expressed and purified following the protocols described previously (16). Mutants of YopO and gelsolin were made by QuikChange site-directed mutagenesis (Stratagene) following the manufacturer's instructions and purified in the same way as the wild-type proteins. Actin was purified from Sf9 cells with GST-tagged gelsolin domains G4–G6, as described previously (32). Rabbit skeletal muscle actin was purified as described previously (33).

### MD Simulations

To understand the dynamics and activation of gelsolin upon serine phosphorylation *in silico*, we carried out 297-ns-long MD simulations of the WT gelsolin (PDB code 3FFN) and phosphorylated gelsolin (Ser-381, Ser-384, and Ser-385). WT and phosphorylated gelsolin were centered in a dodecahedron box and solvated using the TIP3P water model and 150 mm KCl to simulate the cytosolic environment. Energy minimization was performed in Gromacs 4.6 (34) using the all-atom CHARMM 36 force field (32, 35) utilizing a steepest descent algorithm. Next, the equilibration of the system was performed in the following two steps: 100-ps canonical ensemble (NVT) and 100-ps isothermal-isobaric ensemble (NPT) simulation with harmonic restraints (force constant, 1 kJ mol^−1^ K^−1^) applied to the backbone atoms of the protein. The MD simulations were carried out for a further 300 ns with the NPT ensemble without any restraints. During all simulations, a 2-fs integration time step was used. The temperature was maintained at 310 K with the V-rescale algorithm (36), and the pressure was coupled at 1 bar by isotropic pressure coupling utilizing the Parrinello-Rahman algorithm (time constant 2 ps, isothermal compressibility of water, 4.5e^−5^ bar^−1^) (37). Long-range electrostatic interactions were calculated by the fourth order particle mesh Ewald (PME) algorithm. All bonds were constrained using a fourth order P-LINCS algorithm. Electrostatic and van der Waal's interactions were cut off at 1 nm, and a dispersion correction was applied. Periodic boundary conditions were implemented in three dimensions. Initial atomic velocities, prior to NVT equilibration, were obtained from Maxwell's distribution at 310 K. Water molecules were constrained by SETTLE. Neighbor lists for non-bonded interactions were updated at every 20th step using a Verlet cutoff scheme utilizing NVIDIA 970 GPU. Trajectories were analyzed by Gromacs tools, PyMOL (38), in-house bash shell, and Python 2.7.4 (python.org) scripts utilizing the NumPy (numpy.org) and Matplotlib (39) libraries.

### In vitro phosphorylation of gelsolin by YopO for mass spectrometry

Gelsolin was incubated with YopO WT and Sf9 actin in a ratio of 5:1:1 for 30 min at 30 °C in kinase buffer (20 mm HEPES, pH 7.6, 1.0 mm ATP, 1 mm DTT, 10 mm MgCl_2_, 2 mm MnCl_2_) for the phosphorylation reaction to take place. Phosphorylated gelsolin was run on a NuPAGE 4–12% BisTris gel (Invitrogen). The protein band corresponding to gelsolin was excised and then subjected to reduction, alkylation, and in-gel digestion via the following protocol as described previously (40). Gel pieces were washed with 50 μl of 50 mm ammonium bicarbonate. Reduction was carried out by covering the gel pieces with 10 mm DTT for 30 min at 56 °C. Alkylation was carried out with 55 mm iodoacetamide for 20 min in the dark at room temperature. 50 μl of 50 mm ammonium bicarbonate was used for washing, and 50 μl of 100% acetonitrile was used for shrinking twice for 10 min. 30 μl of 13 ng/μl sequencing-grade trypsin (Promega) was added to each well for 30 min at 4 °C before 25 mm ammonium bicarbonate was added to cover the gel pieces. Samples were incubated overnight at 37 °C. Supernatants containing peptides were cleared by centrifugation. 20 μl of 5% formic acid was added to each well followed by 20 μl of 100% acetonitrile for peptide extraction. Both steps were repeated.

### LC/MS analysis

LC/MS analysis was performed by modifying the protocol as described in Ref. 40. Vacuum-dried samples were reconstituted in 0.1% formic acid and analyzed using a nano-HPLC coupled to an LTQ Orbitrap classic (Thermo Fisher Scientific). Peptides were trapped onto a C18 pre-column and separated on an analytical column using a 4-h gradient ranging from 2 to 40% acetonitrile and 0.1% formic acid, followed by a 5-min gradient ranging from 40 to 80% acetonitrile and 0.1% formic acid. Survey full scan MS spectra (*m*/*z* 310–1400) were acquired with a resolution of *r* = 60,000 at *m*/*z* 400, an automatic gain control (AGC) target of 1e^6^, and a maximum injection time of 1000 ms. The 10 most intense peptide ions in each survey scan with an ion intensity of >2000 counts and a charge state of ≥2 were isolated sequentially to a target value of 5000 and fragmented in the linear ion trap by collision-induced dissociation using a normalized collision energy of 35%. A dynamic exclusion was applied using a maximum exclusion list of 500 with one repeat count, repeat duration of 45 s, and exclusion duration of 30 s.

### Mass spectrometry data processing and database searches

Data were processed using MaxQuant (Version 1.3.0.5) (24) against Uniprot 2014-04 human databases containing 262 commonly observed contaminants. Database searches were performed with tryptic specificity allowing a maximum of two missed cleavages and two labeled amino acids as well as an initial mass tolerance of 6 ppm for precursor ions and 0.5 Da for fragment ions. Cysteine carbamidomethylation was searched as a fixed modification, and *N*-acetylation, oxidized methionine, and phosphorylated serine/threonine/tyrosine were searched as variable modifications. Maximum false discovery rates were set to 0.01 for both proteins and peptides. Proteins were considered identified when supported by at least one unique peptide with a minimum length of seven amino acids. The best scoring scan for each detected phosphorylation site with an Andromeda Score of ≥40 were validated manually.

### In vitro γ-^32^P phosphorylation assay

YopO WT (2 μm) was incubated with different gelsolin mutants (6 μm) and adjusted to a final volume of 10 μl with buffer G (2 mm HEPES, pH 7.6, 0.2 mm ATP, 0.5 mm DTT, 0.1 mm CaCl_2_, 1 mm NaN_3_). The kinase reaction was initiated by the addition of an equal volume of 2× kinase buffer supplemented with 5 μCi of [γ-^32^P]ATP. The phosphorylation reactions were carried out for 30 min at 30 °C and terminated by the addition of SDS-PAGE buffer. The samples were heated for 5 min at 95 °C and subjected to SDS-PAGE followed by Coomassie Blue staining. Gels were destained, and radioactivity in the dried SDS-polyacrylamide gels was imaged by exposure to X-ray film.

### Pyrene actin depolymerization assay

The actin depolymerization assay was performed as described previously (22). 10% pyrene-labeled G-actin (12 μm) was incubated with buffer F (50 mm KCl, 0.2 mm ATP, 2 mm MgCl_2_, 0.5 mm DTT, 1 mm EGTA, and 50 mm HEPES, pH 7.5) in 96-well flat-bottomed plates (Corning, NUNC) for 30 min to allow the formation of F-actin. Reactions were equilibrated for 1 h with the required calcium concentrations before 6 μm of the WT gelsolin or mutant proteins were added to a final reaction volume of 100 μl. Fluorescence intensities were measured kinetically with excitation and emission wavelengths of 365 and 407 nm, respectively, using a Safire^2^ fluorimeter (TECAN).

### Actin nucleation assay

To test the nucleation activities of the gelsolin phosphomimetic mutants, 10% pyrene-labeled G-actin (2 μm) was incubated with 50 nm of respective gelsolin proteins for 30 min in the presence of 0.1 mm CaCl_2_ in buffer G in a final volume of 90 μl in 96-well black flat-bottomed plates (Corning, NUNC). 10 μl of 10× KMEI (500 mm KCl, 10 mm MgCl_2_, 10 mm EGTA, 100 mm imidazole HCl, pH 7.0) was added to each reaction before measurement of the fluorescence intensity at excitation and emission wavelengths of 365 and 407 nm, respectively, with a Safire^2^ fluorimeter (TECAN).

### Actin sedimentation assay

Gelsolin was incubated with Sf9 actin and YopO WT or YopO KD in the ratio (2:1:1) for 30 min at 30 °C in kinase buffer to allow phosphorylation to take place. To test the binding of phosphorylated/non-phosphorylated gelsolin to F-actin, various concentrations of the protein (0.0–0.3 μm) were incubated with F-actin (4 μm) in 50 μl of buffer F for 30 min before centrifugation for 30 min at 100,000 × *g* using a TLA120.1 rotor in a Beckman Optima Max ultracentrifuge. WT gelsolin and the PM5 mutant were also tested for their ability to bind F-actin, following the same procedure. The supernatant of each of these experiments was removed and analyzed as the soluble fraction. The pellet was washed by addition of 50 μl of buffer F followed by centrifugation for 30 min at 100,000 × *g*. The supernatant of the second run was discarded, and the pellet was resuspended in 50 μl of buffer F for analysis of the pellet fraction. 15 μl each of the supernatant and resuspended pellet were analyzed by SDS-PAGE to determine the fraction of actin that moved to the soluble fraction indicating severing by gelsolin.

## Author contributions

P. S. and W. L. L. conducted the bulk of the experiments, analyzed the results, and wrote the paper. S. W. conducted mass spectrometry experiments and analyzed the data. U. G. conducted molecular dynamics simulations. K. D. provided technical assistance for the expression and purification of mutant proteins for the revised manuscript. J. G., J. M. G., K. S., and R. C. R. conceived and directed the project.

## Supplementary Material

Supplemental Data

## Footnotes

6 The abbreviations used are:

FcγR: Fc-γ receptor
PIP_2_: phosphatidylinositol 4,5-bisphosphate
GDI: guanidine nucleotide dissociation inhibitor
PDB: Protein Data Bank
BisTris: 2-[bis(2-hydroxyethyl)amino]-2-(hydroxymethyl)propane-1,3-diol
TM: triple mutation
DM: double mutation
MD: molecular dynamics.

## Note added in proof

In the version of this article that was published as a Paper in Press on March 9, 2017, a reference was inadvertently omitted. This error has now been corrected.
