# Supplementary material for: Yersinia effector protein (YopO)-mediated phosphorylation of host gelsolin causes calcium-independent activation leading to disruption of actin dynamics
Source: J Biol Chem. 2017 Mar 9;292(19):8092–100. doi: 10.1074/jbc.M116.757971 (PMC5427284; doi:10.1074/jbc.M116.757971)
Supplement: Supplemental Data [file supp_292_19_8092__index.html]

Yersinia Effector Protein YopO mediated Phosphorylation of Host Gelsolin Causes Calcium-Independent Activation leading to Disruption of Actin Dynamics — Yersinia effector protein (YopO)-mediated phosphorylation of host gelsolin causes calcium-independent activation leading to disruption of actin dynamics — YopO/YpkA activates gelsolin through phosphorylation — Supplemental Data 

# *Yersinia* effector protein (YopO)-mediated phosphorylation of host gelsolin causes calcium-independent activation leading to disruption of actin dynamics

## Supplemental Data

- Supp Data (.docx, 740 KB) - This file contains allowable supplemental data to be published online with article.
