# Supplementary material for: Yersinia effector protein (YopO)-mediated phosphorylation of host gelsolin causes calcium-independent activation leading to disruption of actin dynamics
Source: J Biol Chem. 2017 Mar 9;292(19):8092–100. doi: 10.1074/jbc.M116.757971 (PMC5427284; doi:10.1074/jbc.M116.757971)
Supplement: Supplemental Data [file 10.1074_M116.757971_jbc.M116.757971-1.docx]

**SUPPLEMENTAL DATA**

*Yersinia* Effector Protein YopO–mediated Phosphorylation of Host Gelsolin Causes Calcium-Independent Activation leading to Disruption of Actin Dynamics

Running title: *YopO/YpkA* activates gelsolin through phosphorylation

**Pavithra Singaravelu^1, 2, †^, Wei Lin Lee^1, †,*,^, Sheena Wee^1, †^, Umesh Ghoshdastider^1^, Ke Ding^1^, Jayantha Gunaratne^1,3^, Jonathan M Grimes^4, 5^, Kunchithapadam Swaminathan^2,*^ and Robert C Robinson^1,6,7^**

^1^Institute of Molecular and Cell Biology, A*STAR (Agency for Science, Technology and Research), Singapore

^2^Department of Biological Sciences, National University of Singapore, Singapore

^3^Department of Anatomy, National University of Singapore, Singapore

^4^Division of Structural Biology, Wellcome Trust Centre for Human Genetics, University of Oxford, United Kingdom

^5^Diamond Light Source Ltd., United Kingdom.

^6^Department of Biochemistry, National University of Singapore, Singapore.

^7^NTU Institute of Structural Biology, Nanyang Technological University, 59 Nanyang Drive, Singapore 636921.

^†^ These authors contributed equally to this work

^*^ To whom correspondence should be addressed. Email: Wei Lin Lee [weilin@smart.mit.edu](mailto:weilin@smart.mit.edu), current address Singapore-MIT Alliance for Research and Technology, Singapore; Kunchithapadam Swaminathan [dbsks@nus.edu.sg](mailto:dbsks@nus.edu.sg), Department of Biological Sciences, National University of Singapore, Singapore.


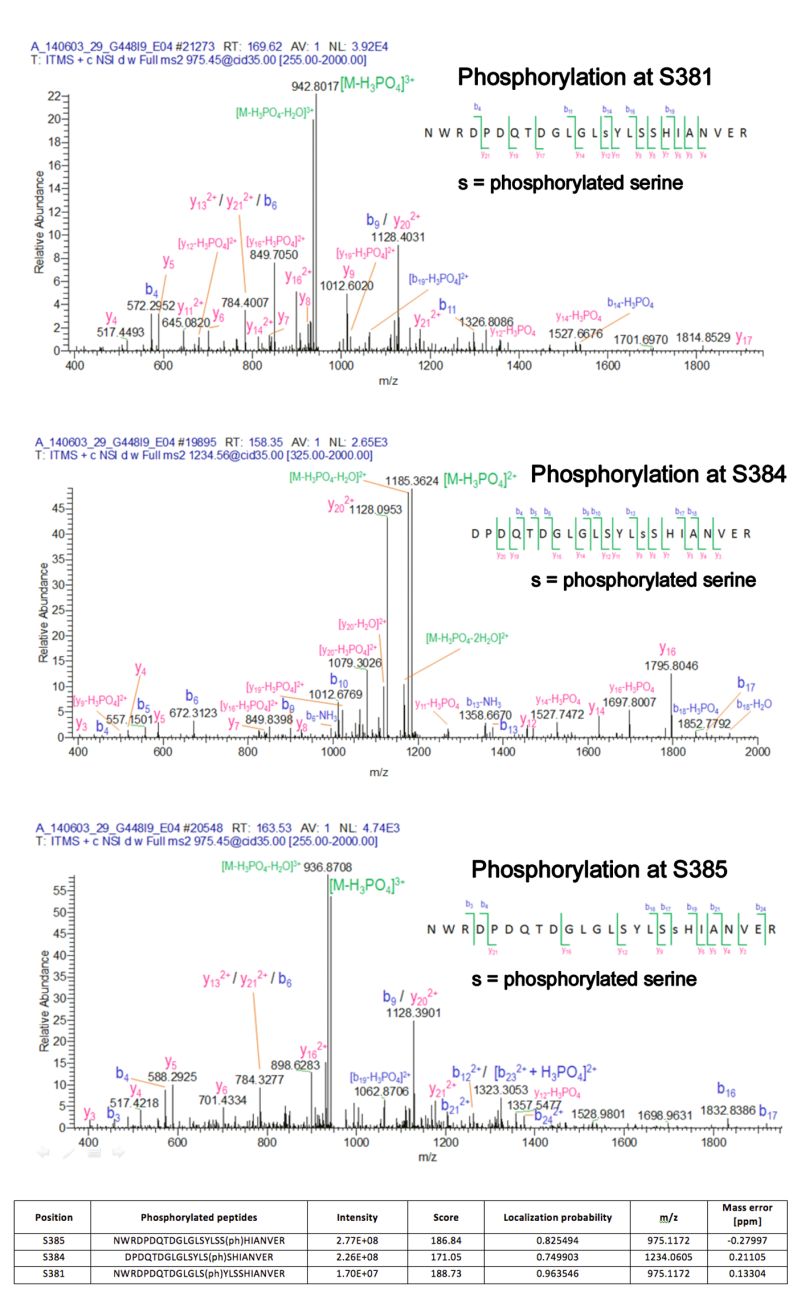


**Figure S1.** ESI-MS spectrum of the phosphorylated gelsolin peptides showing phosphorylation sites identified at Ser381, Ser384 and Ser385.


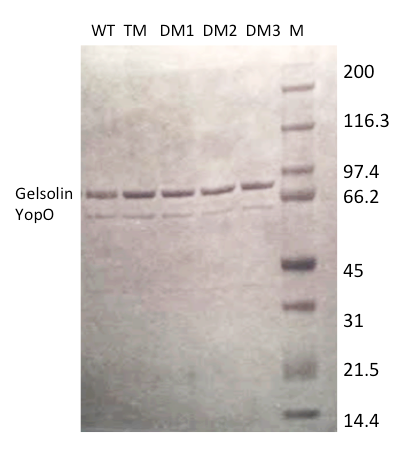


**Figure S2.** Commassie stained SDS-PAGE corresponding to Fig. 1B. M denotes molecular weight marker. WT (wild type gelsolin), [TM, DM1, DM2, DM3] – phospho-deletion gelsolin mutants.

| **Phosphomimetic mutants** | **Original sequence of the linker region** | **Mutations** |
| --- | --- | --- |
| DM1 | S_381_YLS_384_S_385_ | AYLAS |
| DM2 |  | SYLAA |
| DM3 |  | AYLSA |
| TM |  | AYLAA |
| PM1 |  | DYLSD |
| PM2 |  | DYLSS |
| PM3 |  | SYLSD |
| PM4 |  | SYLDS |
| PM5 |  | DYLDD |

**Table 1.** List of gelsolin mutants used in the study.

|  | **Interacting residues** | **Distance (Å)** |
| --- | --- | --- |
| **Ser381** | Asp371 | 2.9 |
|  | Lys363 | 3.6 |
|  | Glu358 | 3.8 |
|  | Gln374 | 4.2 |
|  | Trp369 | 4.7 |
| **Ser385** | Val74 | 4.0 |
|  | Tyr133 | 4.1 |
|  | Lys72 | 4.3 |
|  | Thr129 | 6.0 |
|  | Asp84 | 6.3 |

**Table 2.** List of residues present in the vicinity of Ser381 and Ser385 in the crystal structure of full-length human cytoplasmic gelsolin and their corresponding distances. Distances between the hydroxyl group of the Serine residues and the interactive atoms of the surrounding residues are represented.

|  | M(avg) (kDa) | Error estimates | Polydispersity (Mw/Mn) | Error estimates | R(avg) (nm) | Error estimates |
| --- | --- | --- | --- | --- | --- | --- |
| WT | 77.5 | 0.20% | 1 | 5.52% | 8.8 | 20.30% |
|  | 77.9 | 0.20% | 1.001 | 5.91% | 8.7 | 8.00% |
| PM5 | 78.1 | 0.20% | 1.004 | 5.43% | 9.4 | 11.20% |
|  | 77.4 | 0.20% | 1 | 4.63% | 9.6 | 7.40% |

**Table 3.** Geometric and hydrodynamic radii of gelsolin WT and PM5 examined by SEC-MALS. M(avg), the uncertainty-weighted average molar mass; Mw, weight-average molar mass; Mn, number-average molar mass; R(avg), uncertainty-weighted average geometric and hydrodynamic radius. The theoretical molecular weight of gelsolin is 80.6 kDa.

| YopO phosphorylation sites  Ser381, Ser384 and Ser385 | DGLGL**S**YL**SS** |
| --- | --- |
| Src phosphorylation site  Tyr382 | DGLGLS**Y**LSS |
| Caspase-3 and granzyme cleavage site Asp376 | **D**GLGLSYLSS |
| Granzyme cleavage site  Leu383 | DGLGLSY**L**SS |

**Table 4.** YopO, Src, caspase-3 and granzyme recognition sites in the linker region between the domains G3 and G4 of gelsolin. The recognition sites are indicated in bold.

|  |  | PHOSIDA prediction | |
| --- | --- | --- | --- |
| Substrate | Identified phosphorylation sites | 100% precision | 95% precision |
| Gelsolin | S381  S384  S385 | **S384**, **S385** | S8, S24, S94, S98, S182, S205, S226, S286, S336, S351, **S381**, **S384**, **S385**, S428, S471, S497, S525, S584, S609, S627, S688, S742 |

**Table 5.** Phosphorylation sites predicted for gelsolin by PHOSIDA. Phosphorylation sites that have been identified in native systems, according to PhosphoSitePlus, are underlined. Phosphorylation sites in bold are those identified in this paper which agree with those predicted by PHOSIDA, above a 95% precision cutoff.

**Materials and Methods – Supplementary**

**SEC-MALS (Size Exclusion Chromatography with Multi-Angle Light Scattering)**

For SEC-MALS experiments, CaCl_2_ was added to WT gelsolin and PM5 to a final concentration of 1 mM for 30 min on ice prior to a 4 fold dilution in 10 mM Hepes (pH 7.6), 100 mM NaCl and 1 mM EGTA. The experiments were then performed at room temperature by loading 0.5 mg of each sample in 200 µl onto a Superdex 200 10/300 GL column with a DAWN HELEOS 8+ MALS detector coupled with an Optilab T-rEX refractometer (Wyatt Technology). The column was eluted with buffer containing 10 mM Hepes (pH 7.6), 100 mM NaCl and 1 mM EGTA. A dn/dc (refractive index increment) value of 0.185 ml mg^−1^ was used and light scattering analysis was performed using the Zimm model. Under these buffer conditions gelsolin should quickly adopt its calcium-free conformation due to removal of calcium by the EGTA in the gel filtration buffer.
